# Effects of behavior change techniques in interventions promoting condom use among youth in the Global North

**DOI:** 10.1371/journal.pone.0328467

**Published:** 2025-09-23

**Authors:** Alcira de Vries, Janneke C. M. Heijne, John B. F. de Wit, Chantal den Daas

**Affiliations:** 1 Centre for Epidemiology and Surveillance of Infectious Disease, Centre for Infectious Disease Control, National Institute for Public Health and the Environment, Utrecht, The Netherlands; 2 Department of Infectious Diseases, Public Health Service of Amsterdam, Amsterdam, Noord-Holland, The Netherlands; 3 Institute for Infection and Immunity, Amsterdam UMC Locatie AMC, Amsterdam, Noord-Holland, The Netherlands; 4 Department of Interdisciplinary Social Science, Utrecht University, Utrecht, The Netherlands; 5 Health Psychology group, Institute of Applied Health Sciences, University of Aberdeen, Aberdeen, Scotland, United Kingdom; University of Salamanca, SPAIN

## Abstract

**Background:**

Declines in condom use among the general young population highlight the need for effective interventions to prevent sexually transmitted infections and unwanted pregnancies. With this study, we aim to examine the relationship between Behavior Change Techniques (BCTs, the active components of interventions) and the effects of condom interventions among youth. We quantify the number of BCTs used in the interventions, assess their alignment with underlying behavioral theory, and evaluate coverage of specified Mechanisms of Action (MoA, underlying process through which behavior may be influenced) within the behavioral theory.

**Methods:**

Face-to-face theory-based interventions aiming to promote condom use among youth were identified in a previous systematic review. Interventions were analyzed using the BCT Taxonomy v.1.0., alignment with theory was determined using the Theories and Techniques tool. Wilcoxon rank-sum tests assessed BCT effectiveness. Spearman’s rank correlation coefficient determined associations between intervention effects and the total number of BCTs, the proportion of BCTs aligned with MoAs, and the proportion of MoA covered by BCTs.

**Results:**

In 21 interventions we identified a median of 3 BCTs (IQR = 1–5) per intervention. BCTs were poorly reported. No grouping of BCTs was associated with more intervention effects on increasing condom use. Neither the proportion of BCTs aligned with the MoAs of the underlying theory in the intervention (median = 85.7%, IQR = 50.0–100%, Spearman’s ρ = −0.09) nor the proportion of MoAs of the underlying theory covered by at least one BCT in the intervention (median = 44.4%, IQR = 25.0–50.0%, Spearman’s ρ = 0.27) were correlated with intervention effects.

**Conclusions:**

This study provides initial insights into the use of BCTs and the application of behavioral theory in theory-based condom promotion interventions targeting the general young population. No associations between the use of BCTs and the intervention effects on condom use were found. Robust conclusions regarding the utilization of BCTs, their alignment with theory, and their effects can only be reached when future research consistently and comprehensively reports the use of BCTs.

## Introduction

Condoms are among the most commonly used forms of contraception worldwide, with an estimated 189 million users globally in 2019 [1]. Condoms are still the only contraceptive method to also effectively reduce the risk of sexually transmitted infections (STIs), including HIV [2]. While biomedical advancements, such as pre-exposure prophylaxis (PrEP), have transformed HIV prevention strategies in Key Populations like men who have sex with men (MSM) [3], condoms remain critical in the prevention of non-HIV STIs like chlamydia and gonorrhea in young heterosexual populations. However, research has demonstrated that condom use among youth has decreased over the past 10–15 years in many high-income countries, including many European countries [4–6]. To illustrate, the proportion of Dutch males (12–24 years) who used a condom during their last one-nightstand decreased from 74% in 2005 to 57% in 2023, for Dutch females this decreased from 85% to 53% [7,8]. Young age is a risk factor for contracting sexually transmitted infections (STIs), and it is hence pivotal to effectively promote condom use among young people [9–11].

Behavioral theories, which have been proposed to explain health behavior and provide insights into ways to change behavior, play an important role in developing health promotion interventions, including interventions aiming to improve condom use [12–17]. Evidence of the added value of using behavioral theory in sexual health behavior interventions is mixed. Though a review of systematic reviews of behavioral HIV prevention interventions has demonstrated stronger effects on condom use in theory-based interventions [18], in our recent systematic review of condom promotion interventions, we did not find a difference between condom use effects of interventions that were based on theory or not [19]. Though the populations of these two studies differ (men who have sex with men versus youth in general), there are likely other factors at play that cause this incongruency in the added value of behavioral theory in sexual health promotion.

An explanation for the contradicting findings on the effects of using behavioral theories in interventions is that behavioral theory is not always well applied. Thus, though a study may report the use of a certain theory to design the intervention, this does not necessarily indicate adequate use of the theory. Research has found substantial variation in how theories are applied in health behavior interventions, which may even be applied improperly, including utilizing methods to change behavior that do not align with the underlying theory of factors that influence behavior [20–22]. This variation in theory application complicates the interpretation of intervention effects, as it makes it difficult to assess the added value of using theory in interventions.

Another complication is that reports of intervention studies often do not provide much detail on the determinants of behavior within a theory that were targeted [20–22]. Information on the mechanisms through which behavior change was expected to occur is also often incomplete [20–22]. The lack of detail in reporting and the variability in theory application hamper both the interpretability and replicability of interventions as well al our understanding of how the utilization of theory in condom interventions affects their effectiveness [23].

To improve the reporting, replication, and implementation of behavior change interventions, the Behavior Change Techniques Taxonomy (BCTTv1) has been developed through an extensive process involving expert consensus and empirical testing [23]. This taxonomy defines behavior change techniques (BCTs) as the active components of an intervention, thus the specific techniques employed to influence behavior. The taxonomy provides an overview of 93 distinct techniques that are categorized into 16 groupings, providing a framework for designing and evaluating interventions. Systematically applying theory in interventions could be accomplished by translating a determinant of behavior that can be changed by targeting it with one or more BCTs.

BCTs are not linked to specific determinants from behavioral theories. However, the determinants of behavior from a theory can inform which BCTs are suitable, based on the Mechanism of Action (MoA) that can change the determinants of behavior [24,25]. The interactive online Theory and Techniques Tool, an online, interactive tool, has been developed to link BCTs with their hypothesized MoAs [25,26]. The tool was developed based on a literature synthesis and expert consensus, and provides a framework to support the development and evaluation of behavior change interventions. To illustrate, if the Theory of Planned Behavior is used to inform an intervention, the BCTs *6.2 Social comparison* and *6.3 Information about others’ approval* can influence the Subjective Norm determinant of this theory through the MoA also called Subjective Norms [25]. Consequently, when the used BCTs are reported correctly, i.e., by explicitly using the taxonomy labels, the reporting of BCTs can provide a detailed understanding of the active content in theory-based interventions. This, combined with the correct application of BCTs as is described in the definition provided in the taxonomy, allows for a better understanding of the intervention and its effects.

For multiple health behaviors, e.g., physical activity, substance use, and sedentary behavior, the use of BCTs in interventions has been evaluated in systematic reviews [27–30]. These studies provide insights on frequently reported BCTs and which BCTs were potentially the most effective in improving the behavior. To our knowledge, two systematic reviews have studied the use of BCTs in condom use interventions [31,32]. One review focused on condom use interventions among adults, which found only one intervention (out of five) to be effective, and that intervention applied theory more extensively [31,33]. Indicators for this more extensive use were the mentioning of the theory, the targeting and measuring of behavioral determinants, and whether the theory was tested or refined [31,33]. The one effective intervention also used more BCTs, compared with the ineffective interventions [31]. The second review focused solely on brief interventions (i.e., one-off interventions of a maximum one hour, not including longer-running programs) to improve sexual health in middle-aged adults and found eight BCTs to be frequently reported in effective interventions [32]. These studies provide some insights into how the use of theory related to the effects of the interventions and on which potentially effective BCTs. However, such studies among young people are lacking, while young people are disproportionally affected by STIs. More research is therefore needed using a larger variety of interventions conducted among youth. This would allow for a better understanding of the use BCTs in interventions among this population and consequently of potentially more effective BCTs.

The aim of the current study is to explore which BCTs are more effective in increasing condom use among young heterosexual people in the Global North and identify whether the total number of BCTs is associated with its effects. This adds to the body of evidence on BCTs in condom interventions and provides further insights into their use, reporting, and effects. We also aim to examine the relationship between the BCTs used in interventions and the specific behavioral theories utilized to develop these interventions. By exploring this relationship, we aim to shed light on (1) the extent to which the selected BCTs align with the intervention’s underlying theoretical frameworks and (2) how this relates to the intervention’s effects. We hypothesize that interventions are more effective at increasing condom use when theory is well translated into BCTS than in interventions where theoretical frameworks are poorly translated. This work may help clarify the observed inconsistencies in the added value of theory in condom promotion interventions.

## Materials and methods

### Search strategy and study selection criteria

This study builds on our previous systematic review of condom interventions among youth [19]. Note that the current study is not a systematic review but reports secondary analyses of data extracted from a previously published systematic review. While we did not perform a new systematic search or screening process, we aim to provide transparency by providing relevant supporting information in the supplementary material, including all data used for the current study (S1–S3 Tables). Since the PRISMA checklist was not fully appropriate, we also used the STROBE checklist to ensure clarity.

The previous systematic review included intervention studies promoting condom use among heterosexual youth in Global North countries published between 2010–2021. The initial search was done through Embase, PubMed, Scopus, PsycINFO, and Web of Science and focused on three main concepts: condom use, the general young population, and interventions. Certain geographical areas and studies that only focused on specific populations, such as sexual minorities or incarcerated youth, were excluded to maintain focus on the general population of young people engaging in penile-vaginal sex. Studies on specific population groups likely not engaging in penile-vaginal sex, for instance, men who have sex with men, were therefore excluded because they primarily center their sexual behavior on same-sex activities; however, we recognize that some individuals in this group may still engage in penile-vaginal sex. Additionally, studies of population groups in particular settings, e.g., college students in general, were included, with the understanding that some members of this population may also not engage in penile-vaginal sex. In the main systematic review we assessed overall intervention effects and compared these for different participant-, intervention- and method characteristics, as well as for the behavioral theory on which the intervention was based, if any. The majority of these interventions were delivered face-to-face (59%) and reported using behavioral theory during development (72%). The full search strategy can be found in S1 Text.

For the current study, we identified BCTs in a subset of studies that reported using behavioral theory to develop the intervention, were delivered through human interaction using a face-to-face mode of delivery as described in the Mode of Delivery Ontology [34] (to ensure comparable modes of delivery), and were implemented intervention programs thus excluding theoretical behavioral experiments (n = 21). To ensure comparability of BCTs across interventions, we focused exclusively on face-to-face interventions, as these made up a sufficiently large sample (49 out of 83) [19]. Interventions delivered through other modes, e.g., digitally, were excluded because they represent a more heterogeneous set of modes of delivery, which would have made cross-study synthesis less comparable. By focusing on face-to-face interventions, we were able to assess the BCTs most commonly implemented in this modality. We only included interventions if there was an explicit mention of the use of behavioral theory. While it would be possible to identify BCTs in interventions that were not explicitly based on theory, the identification of the Mechanisms of Action that the intervention aims to target would then have to be inferred based on the content of the intervention, which includes the BCTs that were used. This would have created a risk of circular reasoning, as we aimed to determine the extent to which the used BCTs were suitable for the MoAs of the used theory and the extent to which the MoAs of the used theory were targeted by appropriate BCTs. Determining theory use on the basis of the measured constructs in a study would also have its limitations; interventions that were not based on theory but did measure one or more constructs would wrongfully be classified as being theory-based. If interventions were described in multiple papers (e.g., focusing on outcomes at different time points), outcome data from all papers were included. Efforts were made to retrieve additional publicly available intervention materials through internet searches using the intervention’s name for additional information. We only found scientific publications.

### Data extraction

To guide the data extraction on BCTs, two authors (AdV and CdD) completed an online training available on www.bct-taxonomy.com. These two authors independently identified BCTs in the papers according to the BCT taxonomy [23], using NVIVO. BCTs were only identified if related to the target behavior, condom use, and if sufficient detail on the employed techniques was provided. We calculated Cohen’s kappa’s to assess inter-rater agreement regarding the identification of the BCTs, adjusted for prevalence and bias effects for each identified BCT [23]. Adjusted kappa’s were calculated using the formulas developed by Byrt et al. [35] We used the following cut-off points for the kappa statistic: < 0.00 poor, 0.00–0.20 slight, 0.21–0.40 fair, 0.41–0.60 moderate, 0.61–0.80 substantial, 0.81–1.00 almost perfect [36]. All coded BCTs for which the kappa was not 1.00 were discussed by the two coders. Disagreements were resolved through discussion between the two coders. The coder that did code a certain BCT provided the cited text where the BCT was coded and explained why this demonstrated the presence of that BCT. The coder that did not code the BCT explained why they did not code this BCT. The coders then evaluated the presence of each of the 93 BCTs against each BCT definition provided in the taxonomy. BCTs in the control/sham intervention conditions were not identified.

### Measures

#### Intervention effects on condom use.

The indicator of intervention effects has been described in our previous systematic review [19]. In short, an overall intervention effect score was calculated for each intervention. For each intervention, we calculated an effect score based on the percentage of comparisons that showed significant increases in condom use. Comparisons are defined as each statistical test between intervention and control or pre- and post-intervention, for all different condom use measures (e.g., condom use during the last sex act and the percentage of condom use past 3 months), subgroups (e.g., boys or girls) or follow-up periods (e.g., 3 months and 6 months). We then calculated this effect score per intervention by dividing the number of effective comparisons by the total number of comparisons. To illustrate, if a study measured condom use during the last sex act as an outcome and showed a significant increase in condom use at 3 months but not at 5 months, the intervention’s effect score would be 1/2 = 50%. This intervention effect score was used since we faced challenges due to the high variability in how condom use was measured and analyzed. Most studies on interventions reported multiple intervention outcomes related to condom use (e.g., an intervention could be effective at baseline compared to 3-month follow-up, but not at baseline compared to 6-month follow-up), and many different condom measures were used, and subsequently also many different statistical analyses were conducted to measure intervention effects. Also, study populations differed among interventions. This heterogeneity in condom measures, populations, and analyses also hindered any meta-analyses.

#### Links between MoAs, BCTs and intervention effects.

Based on the Theory and Techniques Tool [25,26], we assessed which BCTs are suitable considering the MoAs in each behavioral theory. To statistically analyze relationships between the application of theory and the effects of the interventions, we composed two variables:


$$BCTs\;aligned\;with\;MoAs=\frac{Number\;of\;reported\;BCTs\;linked\;to\;one\;or\;more\;MoA\;of\;the\;reported\;theory}{Total\;number\;of\;reported\;BCTs\;in\;the\;intervention}$$

$$MoA\;coverage=\frac{Number\;of\;MoA\;that\;are\;targeted\;by\;one\;or\;more\;appropriate\;BCTs}{Total\;number\;of\;MoA\;of\;the\;theory}$$

These variables provide information on both the extent to which the used BCTs are linked to the MoAs and the extent to which determinants specified by behavioral theories are addressed by BCTs. Interventions could be designed based on more than one behavioral theory, thus the variables *BCTs aligned with MoAs* and *MoA coverage* were based on all MoAs of the underlying theories.

### Data analyses

To explore which groupings of BCTs were associated with higher intervention effects, we performed univariate Wilcoxon rank-sum tests per grouping, thus comparing interventions that used at least one BCT from a certain grouping (i.e., the higher-level category to which a BCT was allocated) with interventions that did not use a BCT from this grouping. We only performed these analyses for groupings of BCTs used in 5 or more interventions. While our initial intention was to examine individual BCTs rather than groupings, the limited number of identified BCTs compelled us to adopt the grouping approach.

Using Spearman’s rank correlation coefficient (Spearman’s ρ), we assessed correlations between 1) the variable *BCTs aligned with MoAs*, 2) the variable *MoA coverage* and 3) the total number of identified BCTs in the intervention, and the intervention effect score. Spearman’s rank correlation coefficient, a non-parametric test, was used as the intervention effect score did not follow a normal distribution and the number of observations was low. We also plotted these correlations in scatterplots. Statistical significance was set at 0.05. Analyses were conducted using STATA SE 17.

## Results

### Study- and intervention characteristics

Starting with the included studies from our previous study, we first excluded studies that did not report any behavioral theory (n = 27), then we excluded interventions that were not delivered face-to-face (n = 26) and lastly, we excluded theoretical behavioral experiments (n = 11). The flowchart of the complete paper selection and inclusion process can be found in S1 Fig. Our study included 21 unique interventions that were described in 16 separate publications. Most of these interventions were conducted in the United States of America, among youth aged up until 24 years old, and were in an educational setting (Table 1). Of all 21 interventions, 8 interventions were reported to have been developed using two behavioral theories, the other 13 interventions used one theory (Table 1). In total, there were nine different behavioral theories used in the interventions. Note that interventions can have applied multiple theories, thus percentages of the reported theory in Table 1 do not add up to 100%. The median value of the intervention effect score for the included interventions was 50%. This indicates that 50% of the tests performed to determine an intervention effect showed a significant positive effect on condom use. S4 Table provides an overview of intervention studies and outcome variables.

**Table 1:** Characteristics of the intervention populations and intervention characteristics.

		n	%
Intervention population characteristics			
Region	USA	15	71%
	Europe	6	29%
Sex	Males	1	6%
	Females	8	50%
	Both	7	44%
Age	< 20	8	44%
	20-24	9	50%
	25-29	1	6%
Intervention characteristics			
Setting	Educational	11	52%
	Healthcare facility	6	29%
	Residential facility	1	5%
	Community facility	3	14%
Duration	Single session/administration	6	33%
	< 6 months	9	50%
	≥ 6 months	3	17%
Delivery format	Group	15	75%
	Individual	5	25%
Reported theory	Aids Risk Reduction model	1	5%
	Health Belief Model	4	19%
	Information-Motivation-Behavioral Skills Model	9	43%
	Social-Cognitive Theory	9	43%
	Self-Efficacy Model	1	5%
	Social Learning Theory	1	5%
	Theory of Planned Behavior	1	5%
	Transtheoretical Model of Behavior Change	1	5%
	Theory of Gender and Power	2	10%

### Identified BCTs

In 20 out of 21 interventions, at least 1 BCT was identified, and we identified a total of 20 different BCTs. In six interventions, 1 BCT was identified, in 4 interventions 2 BCTs were identified, and in 10 interventions 3 or more BCTs were identified. The median number of identified BCTs per intervention was 3 (IQR: 1−5), and the maximum number of BCTs was 8. BCTs from 11 different groupings were identified in the interventions (Table 2). We calculated adjusted kappa’s for the 24 initially identified BCTs, which ranged from −0.05–0.90. The BCT *9.1 Credible source* was misinterpreted by one coder, and therefore systematically misidentified, resulting in poor agreement (kappa = −0.05). Of the other identified BCTs, fair agreement was found for 4, moderate agreement for 1, and substantial agreement for 5; agreement was near perfect for 13 BCTs. The two coders discussed disagreements in the identified BCTs. The majority of disagreements was a result of the paper describing the intervention content/techniques in insufficient detail. For example, in an intervention with ‘information about STI/HIV’ it is likely that the use of condoms (our target behavior) to protect against STI/HIV was discussed, but since this link to the target behavior was not explicitly reported, the BCT could not be identified. After discussing such disagreements, some previously identified BCTs were deleted, resulting in a total of 20 different identified BCTs in the interventions.

**Table 2:** Summary of BCTs, theory use and outcomes.

Identified BCTs, and per BCT grouping the values of intervention effect score, *BCTs aligned with MoA* and *MoA coverage*.							Wilcoxon Rank-Sum tests	
BCT grouping	Grouping of BCT identified in interventions (n)	BCT	BCT identified in interventions (n)	BCTs aligned with MoAs (median)	MoA coverage (median)	Intervention effect score (median)	Z-score	P-value
1. Goals and planning	4	1.2 Problem solving	2	67.5	58.3	75.0%	–	–
		1.4 Action planning	1					
		1.7 Review outcome goal(s)	1					
2. Feedback and monitoring	2	2.1 Monitor of behavior by others without feedback	1	87.5	37.5	50.0%	–	–
		2.7 Feedback on outcome(s) of behavior	1					
3. Social support	5	3.1 Social support (unspecified)	5	50.0	50.0	60.0%	−0.55	0.58
4. Shaping knowledge	12	4.1 Instruction on how to perform the behavior	12	92.9	50.8	90.0%	−0.62	0.54
		4.4 Behavioral experiments	1					
5. Natural consequences	15	5.1 Information about health consequences	14	85.7	50.0	90.0%	−0.16	0.87
		5.2 Salience of consequences	2					
		5.3 Information about social and environmental consequences	2					
		5.4 Monitoring of emotional consequences	1					
		5.6 Information about emotional consequences	1					
6. Comparing of behavior	6	6.1 Demonstration of the behavior	6	80.4	58.3	50.0%	0.36	0.72
		6.3 Information about others’ approval	1					
7. Associations	1	7.7 Exposure	1	75.0	44.4	100.0%	–	–
8. Repetition and substitution	7	8.1 Behavioral practice/rehearsal	7	75.0	57.1	100.0%	−0.38	0.70
9. Comparison of outcomes	3	9.1 Credible source	3	60.0	50.0	33.3%	–	–
12. Antecedents	7	12.5 Adding objects to the enviroment	7	60.0	57.1	60.0%	0.04	0.97
13. Identity	2	13.2 Framing/reframing	2	80.4	61.1	100.0%	–	–

### Effective BCTs

**Fig 1:**
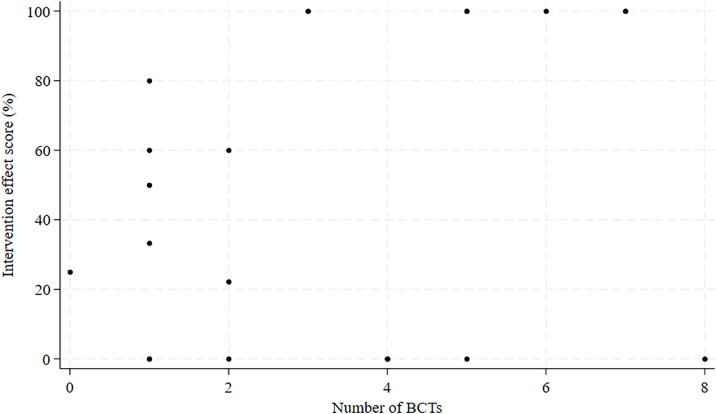
Scatterplot of the total number of identified BCTs in an intervention and the intervention effect score.

We analyzed the association between the total number of identified BCTs in the intervention and the effects of the interventions. We found a Spearman’s ρ of 0.16 (p = .48), indicating no significant correlation (Fig 1). Using Wilcoxon Rank-Sum tests, we assessed whether specific groupings of BCTs were associated with stronger intervention effects than interventions that did not use BCTs of this grouping. The resulting z-scores, which reflect the strength and direction of the association, were close to zero, indicating weak effects. None of these analyses revealed significant effects, indicating no statistically significant differences in the effects of interventions that did or did not use BCTs from a certain grouping (Table 2). Note that z-scores and p-values for BCT groupings identified in <5 interventions are not presented in Table 2. S1 Table displays the identified BCTs and *BCTs aligned with MoAs*, underlying MoAs and *MoA coverage,* and the effects of the intervention in increasing condom use.

### Links between MoAs, BCTs, and intervention effects

**Fig 2:**
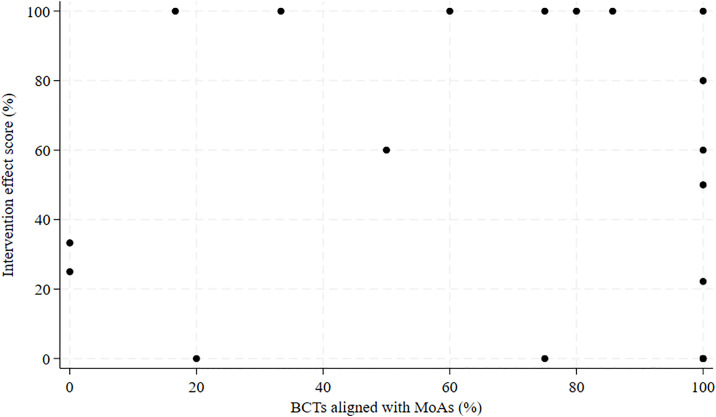
Scatterplot of BCTs aligned with MoAs and the intervention effect score.

For the variable *BCTs aligned with MoAs*, the median was 85.7% (IQR: 50.0–100%), indicating that the majority of BCTs fit with the applied theory. When analyzing the correlation between the percentage of *BCTs aligned with MoAs* and the intervention effects, we found a Spearman’s ρ of −0.09 (p = .71), indicating that there was no significant correlation (Fig 2).

**Fig 3:**
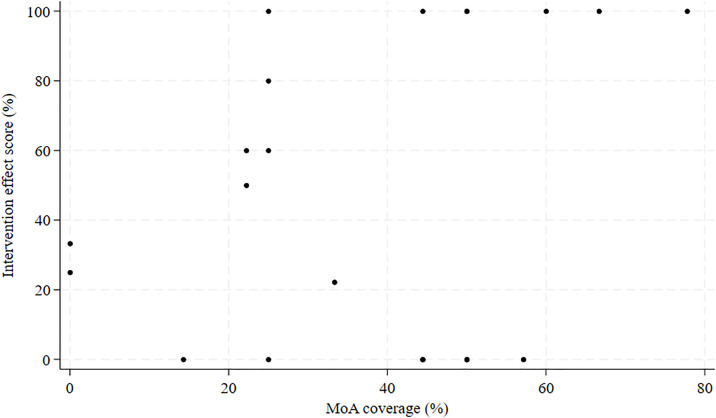
Scatterplot of MoA coverage and the intervention effect score.

The median value of the percentage of *MoA coverage* was 44.4% (IQR: 25.0–50.0%), indicating that few MoAs of the theories used in the interventions were covered by BCTs. When analyzing the correlation between *MoA coverage* and the intervention effects, we found a Spearman’s ρ of 0.27 (p = .24), indicating no significant correlation (Fig 3).

## Discussion

In this study, we examined the use of BCTs and the application of theory in interventions aiming to promote condom use among the general young population. We observed inadequate reporting of BCTs, as the descriptions provided were frequently implicit or lacked sufficient detail to enable accurate identification of the interventions as incorporating specific BCTs. Our study did not find evidence for interventions to be more effective at increasing condom use among youth when BCTs from specific groupings were used or when the underlying theory was well translated into the interventions. We also found that the majority of identified BCTs targeted the underlying theory.

Our study found no specific grouping of BCTs to be associated with more effective interventions. These groupings are based on BCTs that are similar in nature and fall into the same grouping. A systematic review of BCTs in brief interventions to prevent HIV, STI and unintended pregnancies found three individual BCTs (*1.2 Problem solving*, *2.2 Feedback on behavior* and *6.1 Demonstration of behavior*) to be unique to effective interventions and a set of five other BCTs were identified in both effective and ineffective interventions [32]. BCT groupings *1.Goals and planning* and *2.Feedback and monitoring* were too small to analyze in our study. We did test grouping *6.Comparing of behavior* which mainly consisted of BCT *6.1 Demonstration of behavior* but did not find BCT grouping *6.Comparing of behavior* to be associated with more effective interventions. De Vasconcelos et al. [32] argue that interventions should include a core set of all the eight identified BCTs. None of the included interventions in our study incorporated all eight of those BCTs, which may also be a result of a likely underestimation of used BCTs due to insufficient detail reported. While expected, this underreporting is worrisome as this hindered our ability to conclude on the use of theory in relation to the intervention effects and on BCTs that may be more effective. Poor reporting of BCTs in behavioral interventions is a known issue, prompting the development of the Behavior Change Technique Taxonomy [23,37–42]. Though the current BCTTv1 has already improved practice [43], further improvements to the taxonomy are currently being made [44].

The percentage of *BCTs aligned with MoAs* in the interventions was not associated with the effects of the interventions. There was no difference in the effects of the intervention on condom use between interventions where the BCTs were well linked to the underlying theory and interventions where the BCTs were poorly linked to the theory. A plausible explanation is that in general, few BCTs could be identified, and that in most interventions with a maximum of three identified BCTs, all BCTs were aligned with MoAs. Also, since the values of *BCTs aligned with MoAs* had limited variation and overall were relatively high, it becomes more difficult to detect associations with the intervention effects.

The majority of BCTs in the interventions were aligned with MoAs related to the behavioral theory used to develop the intervention. This, however, does not necessarily indicate a good translation of theory into practice. Often, these BCTS came from grouping *4. Shaping knowledge* and *5. Natural consequences* and translated into action by providing information on condom use and the consequences of not using condoms. These BCTs are linked to multiple MoAs (e.g., *knowledge*, *skills, beliefs about consequences),* which are included in the majority of theories reported in the studied interventions. Looking more in-depth into these BCTs that focus on information, we may find their high prevalence in condom use interventions to stem from a common-sense approach: to change behavior, one should provide information on what to do and why. Contrary to many other health behaviors with various motivations (e.g., physical exercise for health or to socialize [45]) condom use primarily revolves around its crucial purpose of preventing STIs and unwanted pregnancies. Moreover, condom use has multiple negative aspects, including reducing intimacy and sexual pleasure [46–48]. It may thus be necessary to inform youth about the preventive purpose of condoms, given that this is the sole motivation for many individuals. Therefore, the fact that many interventions provide knowledge on condom use may be a logical finding given the nature of the behavior.

Our study found no association between MoA coverage and intervention effects, suggesting that interventions with well-targeted MoAs did not yield better results than interventions with poorly targeted MoAs. Behavioral theories consist of multiple determinants reflecting different MoAs that can subsequently be influenced by multiple BCTs. While higher MoA coverage might therefore be expected to bring about more profound changes in behavior, our study identified generally low MoA coverage in interventions. This likely results from poor BCT reporting and insufficiently explicit intervention descriptions, thus resulting in an underestimation of used BCTs and covered MoAs. Interventions with lower MoA coverage did indeed have lower numbers of identified BCTs than interventions with higher MoA coverage. It is also possible that MoA coverage simply is low, irrespective of the reporting of BCTs. In that case, interventions only focused on parts of the underlying behavioral theory, thus only a selection of determinants that influence behavior. This could serve as an explanation why in our previously conducted systematic review we could not find a difference in intervention effects between interventions that used behavioral theory and interventions that did not [19].

Interventions with higher MoA coverage more frequently reported the use of the Health Belief Model (HBM)[12,49]. Behavioral determinants of the HBM include perceived susceptibility, perceived severity and perceived benefits. These determinants can be influenced by the MoAs *perceived severity* and *beliefs about consequences* [50]. The majority of interventions included BCTs from the groupings *Shaping knowledge* and *Natural consequences,* which contain BCTs that are linked to the MoAs *perceived severity* and *beliefs about consequences*. In the interventions with higher MoA coverage that reported use of the HBM, only the *perceived severity* and *beliefs about consequences* MoA were covered by BCTs, while the other MoAs (*motivation* and *behavioral regulation*) that can influence the remaining behavior determinants were untargeted. Since these interventions provided limited information on the selection and application of theory, it remains uncertain whether the higher *MoA coverage* was a result of carefully selected BCTs based on theory, or whether the higher *MoA coverage* was a positive but unintended result of using commonly used BCTs that happened to be a good fit with the Health Belief Model.

In the current study, the median value of the intervention effect score for the included interventions was 50%. This means that 50% of the analyses conducted to evaluate the intervention’s effectiveness demonstrated a significant positive impact on condom use. This is higher than the 33.3% we found for the interventions included in our previous work, a systematic review of different condom interventions [19]. This difference may be a result of the current inclusion criteria. For the current study, we focused on face-to-face interventions with a reported use of theory, whereas the systematic review included a broader range of interventions, not limiting to certain modes of delivery of the use of theory. While the systematic review could not find indications that theory-based interventions were more effective in increasing condom use, the median intervention effect score of 50% in the current study suggests that theory-based face-to-face interventions may be more effective. However, statistically examining this was beyond the scope of the current study, and therefore no hard conclusions can be drawn.

A strength of our study is that we not only examined BCTs aligned with MoAs but also assessed MoA coverage. To illustrate, if an intervention used only one BCT that aligned with a MoA, it would incorrectly indicate a good translation of theory. By assessing the extent to which MoA coverage is achieved, our study offers a more comprehensive view of theory application. In addition, since we studied the effects of the interventions in our previous systematic review, we also were able to analyze the use of BCTs and the application of theory in combination with the intervention’s ability to improve condom use. This is an important addition to the currently limited research on the use and effects of BCTs in the context of condom use among youth.

Important to note, that due to high levels of heterogeneity of studies in, e.g., condom measures and analyses, a meta-analytic approach was not feasible. Meta-analyses based on multiple different effect sizes and including numerous moderators would not have yielded meaningful results. Instead, our intervention effect outcome focused on whether interventions significantly increased condom use, regardless of effect size. That is, small and significant increases in condom use were treated the same as larger increases. Small but not statistically significant increases in condom use were not considered. Note that in the prior systematic review we found no indication that the type of outcome measure (e.g., short-term outcomes) were associated with stronger intervention outcomes. However, we acknowledge that our integration of timepoints may introduce a bias favoring positive outcomes for studies that only assessed short-term effects and did not evaluate long-term effects, compared to studies that assessed both short-term- and long-term effects but observed null effects in the long-term. While this metric for effectiveness offers a standardized approach, it inherently risks overestimating positive outcomes in such cases, and this limitation should be kept in mind when interpreting the findings.

Another point worth noting is that condom interventions may have been evolving in recent years due to the increasing availability and uptake of PrEP, which is especially relevant to Key Populations like MSM. Since our study focused on general heterosexual youth in the Global North, condom interventions likely are minimally impacted by recent PrEP advancements. Focusing on the Netherlands, we see that zero heterosexual men and only 29 female PrEP users were included in the National PrEP pilot program [51], and 102 new HIV diagnoses were made among heterosexual people with heterosexual sex as the main route of transmission in 2023 [52]. Additionally, we acknowledge that our decision to exclude certain populations, such as sexual minorities, may have introduced bias into our findings. By excluding these groups, the study might have lost insights into condom use behaviors in populations whose sexual behaviors may not always align neatly with our assumed focus on penile-vaginal sex. This limitation should be considered in the interpretation of the results and their generalizability. Our findings may therefore only be translated to similar contexts and should not be applied to populations with different STI epidemiology and sexual health promotion strategies. Also, findings only apply to face-to-face interventions and future research could address other types of interventions.

Furthermore, we faced limitations in accurately identifying BCTs due to the lack of detail provided in the papers, precluding a comprehensive analysis of technique usage and theory application. While we tried to retrieve additional intervention materials, we only found published studies, likely resulting in an underestimation of identified BCTs [53]. Also important to note is that we only included interventions when the used theory was explicitly mentioned. This may therefore have excluded interventions that were developed based on theory but did not explicitly state this. Also, we based the theory’s MoAs based on which MoAs are included in the theory rather than which MoAs of the theory were targeted in the study. Some studies may have used only a selection of the theory’s MoAs based on the specific needs of their study population. This may have resulted in an underestimation of MoA coverage and of the degree to which BCTs aligned with the theory’s MoAs. Additionally, our study only included interventions using a face-to-face delivery, to have a comparable mode of delivery across interventions. Future research could explore BCTs in interventions delivered through other modes, as this may yield different effects. Also, since we included studies published from 2010 to 2021, the most recent interventions are not presented in our study. However, the aim of the current study was not to provide the most up-to-date overview of interventions, but rather to delve deeper into the use of theory and BCTs in a solid sample of theory-based face-to-face interventions. Also, by excluding interventions that were implemented during the COVID-19 pandemic, we ensure that our findings are generalizable to normal circumstances.

## Conclusions

Reversing the declining trend of condom use among the general young population over the past 10–15 years in many European countries remains a challenge. Developing and reporting interventions is a complex and multifaced endeavor, and condom use is not an easy behavior to change. Higher numbers of BCTs were not associated with more intervention effects, nor were specific groupings of BCTs. Neither BCT alignment with Mechanisms of Action nor mechanism of Action coverage were associated with the effects of the interventions in increasing condom use. Sufficient detail on the used BCTs should be provided in intervention evaluation reports, as this is crucial for the interpretation, replication and improvement of interventions. More research using well-reported studies is essential to enable the formulation of concrete practical recommendations. Nevertheless, our study offers initial insights into the utilization of BCTs and theory application in theory-based condom promotion interventions targeting youth.

## Supporting information

S1 Fig Flowchart of the paper selection and inclusion process of the main systematic review and the current study. (DOCX)

S1 Table All papers included in the main systematic review and the reason for exclusion from the current study. (DOCX)

S2 Table Extracted and used data for the current study. (XLSX)

S3 Table All records identified in the database search for the main systematic review. (XLSX)

S4 Table Overview of intervention studies and outcome variables. (XLSX)

S1 Text Search strategy for each of the used databases. (DOCX)

S1 File STROBE checklist. (DOCX)

S2 File PRISMA checklist. (DOCX)
