# Supplementary material for: Effects of behavior change techniques in interventions promoting condom use among youth in the Global North
Source: PLoS One. 2025 Sep 23;20(9):e0328467. doi: 10.1371/journal.pone.0328467 (PMC12456814; doi:10.1371/journal.pone.0328467)
Supplement: S1 Fig — (DOCX) [file pone.0328467.s001.docx]

Figure S1,  Flowchart of the paper selection and inclusion process of the main systematic review and the current study.


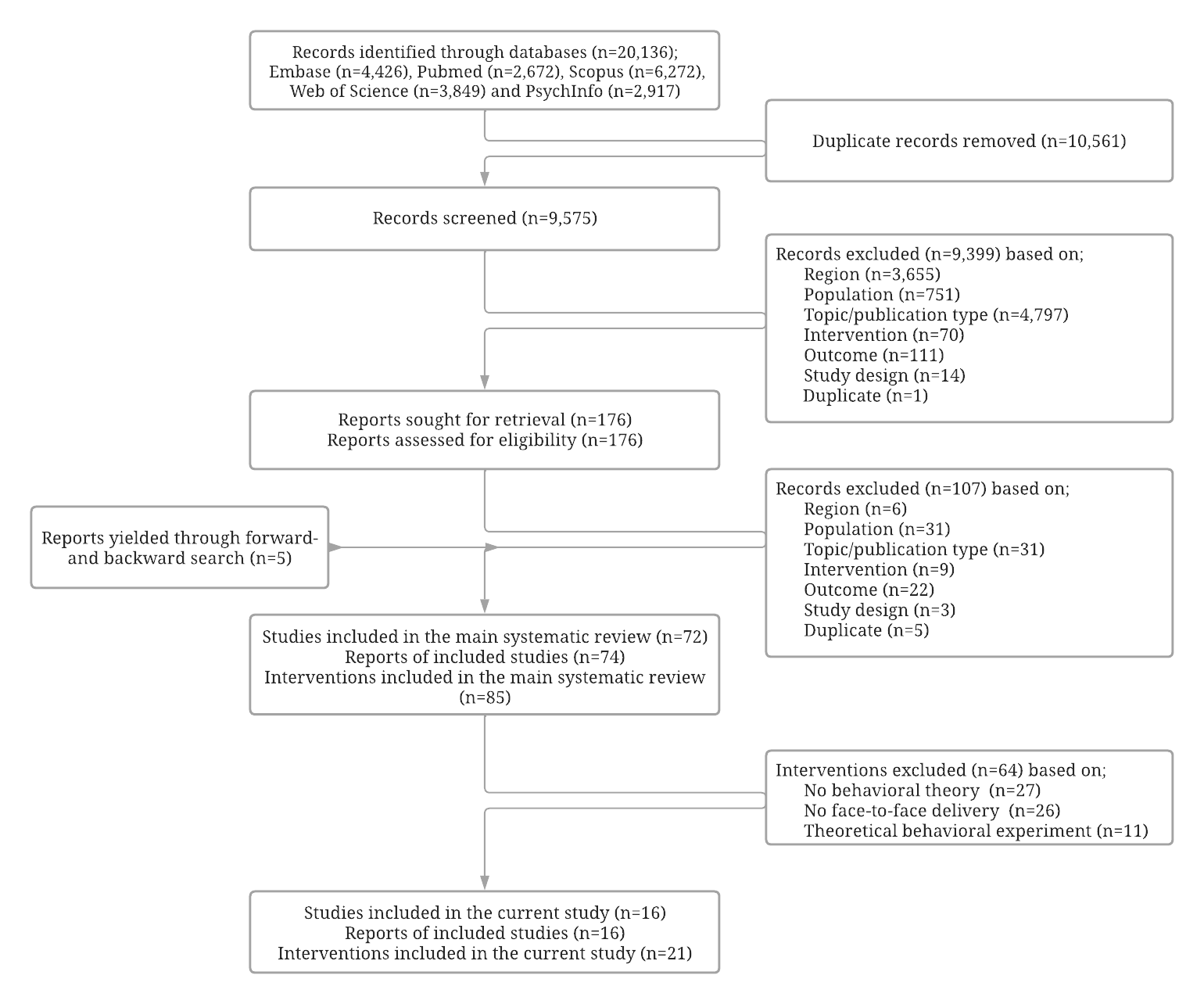


Adapted from de Vries et al (1)

1. de Vries A, den Daas C, Willemstein IJM, de Wit JBF, Heijne JCM. Interventions promoting condom use among youth: a systematic review. Journal of Adolescent Health. 2024;74(4):644-56.
