# Supplementary material for: Effects of behavior change techniques in interventions promoting condom use among youth in the Global North
Source: PLoS One. 2025 Sep 23;20(9):e0328467. doi: 10.1371/journal.pone.0328467 (PMC12456814; doi:10.1371/journal.pone.0328467)
Supplement: S1 Table — (DOCX) [file pone.0328467.s002.docx]

S1 Table, Overview of intervention studies and outcome variables

| Author | Intervention name/condition | Theory | Coded BCTs (n) | BCTs aligned with MoAs (%) | MoA in theory/theories (n) | MoA coverage (%) | Intervention effect score (%) | # Tests for an effect |
| --- | --- | --- | --- | --- | --- | --- | --- | --- |
| Aronson et al(1) | Brothers Leading Healthy Lives (BLHL) | IMBS | 4 | 100% | 9 | 67% | 100% | 2 |
| Berenson et al(2) | Clinic-based + phone call | HBM | 6 | 17% | 4 | 50% | 100% | 1 |
| Berenson et al(2) | Clinic-based | HBM | 5 | 20% | 4 | 50% | 0% | 1 |
| Calloway et al(3) | Playing it Safe | SCT + HBM | 1 | 100% | 7 | 14% | 0% | 1 |
| Cunha-Oliveira et al(4) |  | IMBS + ARRM | 0 | 0% | 11 | 0% | 25% | 4 |
| DiClemente et al(5) | Horizons + GMET | SCT | 2 | 50% | 4 | 25% | 100% | 3 |
| DiClemente et al(5, 6) | Horizons | SCT | 1 | 100% | 4 | 25% | 33% | 9 |
| Ferrer et al(7) | Social cognitive emotion | IMBS | 7 | 86% | 9 | 78% | 100% | 2 |
| Ferrer et al(7) | Social cognitive | IMBS | 4 | 100% | 9 | 44% | 0% | 2 |
| Gimenez-Garcia et al(8) | Peer-led | IMBS | 1 | 100% | 9 | 22% | 60% | 10 |
| Gimenez-Garcia et al(8) | Expert-led | IMBS | 1 | 100% | 9 | 22% | 50% | 10 |
| Kelsey et al al(9) | Safer Sex Intervention | SCT + TTM | 8 | 75% | 4 | 50% | 0% | 3 |
| Morales et al(10, 11) | Cuidate | SCT + TPB | 4 | 100% | 7 | 57% | 15% | 13 |
| Morales et al(10, 11) | Compas | IMBS + SLT | 2 | 100% | 9 | 44% | 25% | 8 |
| Morrison-Beedy et al(12) |  | IMBS | 2 | 100% | 9 | 33% | 22% | 18 |
| Nebot et al(13) |  | SCT | 2 | 50% | 4 | 25% | 60% | 5 |
| Sapiano et al(14) | Sisters Informing Sisters about Topics on Aids (SISTA) | SCT + TGP | 3 | 100% | 4 | 25% | 100% | 3 |
| Sieving et al(15-17) | Prime Time | SCT | 1 | 100% | 4 | 25% | 80% | 5 |
| Wingood et al(18) |  | SCT + TGP | 1 | 0% | 4 | 0% | 33% | 3 |
| Yarber et al(19) | Kinsey Institute Home-based Exercises for Responsible Sex (KIHERS) | IMBS | 7 | 75% | 9 | 44% | 100% | 1 |
| Zellner et al(20) | Color It Real | HBM + SEM | 5 | 60% | 5 | 60% | 100% | 1 |

* Abbreviations: Information-motivation-behavior-Skills Model (IMBS), Health Belief Model (HBM), Social Cognitive Theory (SCT), Social Learning Theory (SLT), AIDS Risk Reduction Model (ARRM), Theory of Gender and Power (TGP), self-Efficacy Model (SEM)

1. Aronson RE, Rulison KL, Graham LF, Pulliam RM, McGee WL, Labban JD, et al. Brothers Leading Healthy Lives: Outcomes from the pilot testing of a culturally and contextually congruent HIV prevention intervention for black male college students. AIDS education and prevention : official publication of the International Society for AIDS Education. 2013;25(5):376-93.

2. Berenson AB. A study of two interventions to increase adherence with oral contraceptives and condom use among adolescents and young adults. Dissertation Abstracts International: Section B: The Sciences and Engineering. 2013;73(11-B(E)):No-Specified.

3. Calloway DS, Long-White DN, Corbin DE. Reducing the risk of HIV/AIDS in African American college students: an exploratory investigation of the efficacy of a peer educator approach. Health Promot Pract. 2014;15(2):181-8.

4. Cunha-Oliveira A, Caramelo F, Patrício M, Camarneiro A, Cardoso S, Pita J. Impact of an educational intervention program on the sexual behaviors of higher education students. Revista de Enfermagem Referência. 2017;IV Série:71-82.

5. DiClemente RJ, Wingood GM, Sales JM, Brown JL, Rose ES, Davis TL, et al. Efficacy of a telephone-delivered sexually transmitted infection/human immunodeficiency virus prevention maintenance intervention for adolescents: a randomized clinical trial. JAMA Pediatr. 2014;168(10):938-46.

6. DiClemente RJ, Rosenbaum JE, Rose ES, Sales JM, Brown JL, Renfro TL, et al. Horizons and Group Motivational Enhancement Therapy: HIV Prevention for Alcohol-Using Young Black Women, a Randomized Experiment. Am J Prev Med. 2021;60(5):629-38.

7. Ferrer RA, Fisher JD, Buck R, Amico KR. Pilot Test of an Emotional Education Intervention Component for Sexual Risk Reduction. Health Psychology. 2011;30(5):656-60.

8. Giménez-García C, Ballester-Arnal R, Gil-Llario MD, Salmerón-Sánchez P. Peer-Led or Expert-Led Intervention in HIV Prevention Efficacy? A Randomized Control Trial Among Spanish Young People to Evaluate Their Role. Health promotion practice. 2018;19(2):277-86.

9. Kelsey M, Walker JT, Layzer J, Price C, Juras R. Replicating the Safer Sex Intervention: 9-Month Impact Findings of a Randomized Controlled Trial. Am J Public Health. 2016;106(S1):S53-s9.

10. Morales A, Espada JP, Orgilés M. A 1-year follow-up evaluation of a sexual-health education program for Spanish adolescents compared with a well-established program. European journal of public health. 2016;26(1):35-41.

11. Espada JP, Escribano S, Morales A, Orgilés M. Two-Year Follow-Up of a Sexual Health Promotion Program for Spanish Adolescents. Evaluation & the Health Professions. 2017;40(4):483-504.

12. Morrison-Beedy D, Jones SH, Xia Y, Tu X, Crean HF, Carey MP. Reducing sexual risk behavior in adolescent girls: Results from a randomized controlled trial. Journal of Adolescent Health. 2013;52(3):314-21.

13. Nebot L, Díez E, Martín S, Estruga L, Villalbí JR, Pérez G, et al. [Effects of a contraceptive counselling intervention in adolescents from deprived neighbourhoods with a high proportion of immigrants]. Gac Sanit. 2016;30(1):43-6.

14. Sapiano TN, Moore A, Kalayil EJ, Zhang X, Chen B, Uhl G, et al. Evaluation of an HIV prevention intervention designed for African American Women: results from the SISTA Community-Based Organization Behavioral Outcomes Project. AIDS Behav. 2013;17(3):1052-67.

15. Sieving RE, McMorris BJ, Beckman KJ, Pettingell SL, Secor-Turner M, Kugler K, et al. Prime time: 12-month sexual health outcomes of a clinic-based intervention to prevent pregnancy risk behaviors. Journal of Adolescent Health. 2011;49(2):172-9.

16. Sieving RE, McRee AL, Secor-Turner M, Garwick AW, Bearinger LH, Beckman KJ, et al. Prime Time: long-term sexual health outcomes of a clinic-linked intervention. Perspectives on sexual and reproductive health. 2014;46(2):91-100.

17. Sieving RE, McRee AL, McMorris BJ, Beckman KJ, Pettingell SL, Bearinger LH, et al. Prime time: Sexual health outcomes at 24 months for a clinic-linked intervention to Prevent pregnancy risk behavior. JAMA Pediatrics. 2013;167(4):333-40.

18. Wingood GM, Diclemente RJ, Robinson-Simpson L, Lang DL, Caliendo A, Hardin JW. Efficacy of an HIV intervention in reducing high-risk human papillomavirus, nonviral sexually transmitted infections, and concurrency among African American women: a randomized-controlled trial. J Acquir Immune Defic Syndr. 2013;63 Suppl 1(0 1):S36-43.

19. Yarber WL, Milhausen RR, Beavers KA, Ryan R, Sullivan MJ, Vanterpool KB, et al. A pilot test of a self-guided, home-based intervention to improve condom-related sexual experiences, attitudes, and behaviors among young women. Journal of American college health : J of ACH. 2018;66(5):421-8.

20. Zellner T, Trotter J, Lenoir S, Walston K, Men-Na'a L, Henry-Akintobi T, et al. Color It Real: A Program to Increase Condom Use and Reduce Substance Abuse and Perceived Stress. Int J Environ Res Public Health. 2015;13(1):ijerph13010051.
