# Supplementary material for: Effects of behavior change techniques in interventions promoting condom use among youth in the Global North
Source: PLoS One. 2025 Sep 23;20(9):e0328467. doi: 10.1371/journal.pone.0328467 (PMC12456814; doi:10.1371/journal.pone.0328467)
Supplement: S1 Text — (DOCX) [file pone.0328467.s006.docx]

**Text S1, Search strategy for each of the used databases**

For the initial systematic review, we used five databases. The search strategy of each is described below. Source: (de Vries et al., 2024)

Embase

'condom use'/exp/mj OR 'condom*':ti,ab OR 'condom'/exp OR 'safe sex'/exp OR 'safe* sex*':ti,ab OR 'safe intercourse':ti,ab OR 'unsafe sex*':ti,ab OR 'unsafe intercourse':ti,ab OR 'protected sex*':ti,ab OR 'protected intercourse':ti,ab OR 'unprotected sex*':ti,ab OR 'unprotected intercourse':ti,ab

'young adult'/exp OR 'young adult*':ti OR 'youth*':ti,ab OR 'young*':ti,ab OR 'student*':ti,ab OR 'student'/exp OR 'teen*':ti,ab OR 'school*':ti,ab OR 'boy*':ti,ab OR 'girl*':ti,ab OR 'aya':ti,ab OR 'adolescent'/exp OR 'adolesc*':ti,ab

'intervention study'/exp OR 'intervention*':ti,ab OR project*:ti,ab OR 'program*':ti,ab OR 'educat*':ti,ab OR 'prevent*':ti,ab OR 'promot*':ti,ab OR 'counsel*':ti,ab OR 'strateg*':ti,ab OR 'communicat*':ti,ab OR 'service*':ti,ab OR 'pilot study'/exp OR 'program development'/exp OR 'program evaluation'/exp OR 'voluntary program'/exp OR 'internet-based intervention'/exp OR 'sexual education'/exp OR 'health promotion'/exp OR 'sex counseling'/exp

#1 AND #2 AND #3 AND [2010-2021]/py NOT 'asia'/exp NOT 'asia*':ti,ab NOT 'africa'/exp NOT 'africa*':ti,ab NOT 'latin america':ti,ab NOT 'south america'/exp NOT 'south america':ti,ab NOT 'abuse':ti,ab NOT 'violence':ti,ab NOT 'latin america'/exp NOT 'abuse'/exp NOT 'violence'/exp NOT 'msm':ti NOT 'ymsm':ti NOT 'men who have sex with men':ti NOT 'sex work*':ti NOT 'prostitut*':ti NOT 'drug*':ti

Pubmed

(condoms[MeSH Terms]) OR (condoms[MeSH Major Topic]) OR (condom[Title/Abstract]) OR (safe sex[MeSH Terms]) OR (safe* sex*[Title/Abstract]) OR (safe intercourse[Title/Abstract]) OR (unsafe sex*[Title/Abstract]) OR (unsafe intercourse[Title/Abstract]) OR (protected sex*[Title/Abstract]) OR (protected intercourse[Title/Abstract]) OR (unprotected sex*[Title/Abstract]) OR (unprotected intercourse[Title/Abstract])

(adult, young[MeSH Terms]) OR (youth*[Title/Abstract] ) OR (young*[Title/Abstract] ) OR (student*[Title/Abstract] ) OR (teen*[Title/Abstract] ) OR (school*[Title/Abstract] ) OR (boy[Title/Abstract] ) OR (boys[Title/Abstract] ) OR (girl*[Title/Abstract] ) OR (aya[Title/Abstract] ) OR (adolescent[MeSH Terms]) OR (adolesc*[Title/Abstract])

(intervention*[Title/Abstract] ) OR (project*[Title/Abstract] ) OR (program*[Title/Abstract] ) OR (educat*[Title/Abstract] ) OR (prevent*[Title/Abstract] ) OR (promot*[Title/Abstract] ) OR (counsel*[Title/Abstract] ) OR (strateg*[Title/Abstract] ) OR (communicat*[Title/Abstract] ) OR (service*[Title/Abstract] ) OR (pilot study[MeSH Terms] ) OR (program development[MeSH Terms] ) OR (program evaluation[MeSH Terms] ) OR (voluntary program[MeSH Terms] ) OR (Internet-based intervention[MeSH Terms] ) OR (sexual education[MeSH Terms] ) OR (health promotion[MeSH Terms] ) OR (sex counseling[MeSH Terms])

("2010/01/01"[Date - Publication] : "3000"[Date - Publication]) NOT (asia[MeSH Terms]) NOT (africa[MeSH Terms]) NOT (latin america[MeSH Terms]) NOT (south america[MeSH Terms]) NOT (central america[MeSH Terms]) NOT (caribbean region[MeSH Terms]) NOT (abuse[Title/Abstract]) NOT (violence[Title/Abstract]) NOT (abuse[MeSH Terms]) NOT (violence[MeSH Terms]) NOT (MSM[Title]) NOT (YMSM[Title]) NOT (men who have sex with men[Title]) NOT (sex work*[Title]) NOT (prostitut*[Title]) NOT (drug*[Title])

#1 AND #2 AND #3

#5 NOT #4

SCOPUS

(TITLE-ABS-KEY (condom*) OR TITLE-ABS-KEY (safe* PRE/1 sex*) OR TITLE-ABS (safe* PRE/1 intercourse) OR TITLE-ABS (unsafe PRE/1 sex*) OR TITLE-ABS (unsafe PRE/1 intercourse) OR TITLE-ABS (protected PRE/1 sex*) OR TITLE-ABS (protected PRE/1 intercourse) OR TITLE-ABS (unprotected PRE/1 sex*) OR TITLE-ABS (unprotected PRE/1 intercourse))

AND

(TITLE-ABS (young PRE/1 adult*) OR KEY (young adult) OR TITLE-ABS(young*)OR TITLE-ABS(youth*)OR TITLE-ABS(student*)OR TITLE-ABS(teen*)OR TITLE-ABS(school*)OR TITLE-ABS(boy*)OR TITLE-ABS(girl*) OR TITLE-ABS(aya)OR TITLE-ABS-KEY(adolesc*))

AND

(TITLE-ABS( intervention* )OR TITLE-ABS( project* )OR TITLE-ABS( program* )OR TITLE-ABS( educat* ) OR TITLE-ABS( prevent* )OR TITLE-ABS( promot* )OR TITLE-ABS( counsel* )OR TITLE-ABS( strateg* )OR TITLE-ABS( communicat* )OR TITLE-ABS( service* ) OR KEY (pilot PRE/1 study ) OR KEY (program PRE/1 development ) OR KEY (program evaluation ) OR KEY (voluntary PRE/1 program ) OR KEY (Internet-based PRE/1 intervention ) OR KEY (sexual PRE/1 education ) OR KEY (health PRE/1 promotion ) OR KEY (sex PRE/1 counseling))

AND

(PUBYEAR > 2009 AND NOT KEY(asia) AND NOT KEY(africa) AND NOT KEY(latin PRE/1 america) AND NOT KEY(south PRE/1 america) AND NOT KEY(central PRE/1 america) AND NOT KEY(caribbean) AND NOT TITLE-ABS-KEY(abuse) AND NOT TITLE-ABS-KEY(violence) AND NOT TITLE(MSM) AND NOT TITLE(YMSM) AND NOT TITLE(men who have sex with men) AND NOT TITLE(sex work*) AND NOT TITLE(prostitut*) AND NOT TITLE(drug*))

Web of science

(TI= (''condom*'' OR ''safe* sex*'' OR ''safe intercourse'' OR ''unsafe sex*'' OR ''unsafe intercourse'' OR ''protected sex*'' OR ''protected intercourse'' OR ''unprotected sex*'' OR ''unprotected intercourse'') )

OR (AB= (''condom*'' OR ''safe* NEAR/1 sex*'' OR ''safe NEAR/1 intercourse'' OR ''unsafe NEAR/1 sex*'' OR ''unsafe NEAR/1 intercourse'' OR ''protected NEAR/1 sex*'' OR ''protected NEAR/1 intercourse'' OR ''unprotected NEAR/1 sex*'' OR ''unprotected NEAR/1 intercourse'') )

OR (KP = (''condom*'' OR ''safe* NEAR/1 sex*'') )

OR (AK = (''condom*'' OR ''safe* NEAR/1 sex*''))

(TI= (''young*'' OR ''youth*'' OR ''student*'' OR ''teen*'' OR ''school*'' OR ''boy*'' OR ''girl*'' OR ''aya'' OR ''adolesc*'') )

OR (AB = (''young*'' OR ''youth*'' OR ''student*'' OR ''teen*'' OR ''school*'' OR ''boy*'' OR ''girl*'' OR ''aya'' OR ''adolesc*'') )

OR (KP= (''young adult*'' OR ''adolesc*''))

OR (AK= (''young adult*'' OR ''adolesc*''))

(TI= (‘‘intervention*’‘ OR project* OR ‘‘program*’‘ OR ‘‘educat*’‘ OR ‘‘prevent*’‘ OR ‘‘promot*’‘ OR ‘‘counsel*’‘ OR ‘‘strateg*’‘ OR ‘‘communicat*’‘ OR ‘‘service*’‘ ) )

OR (AB= (‘‘intervention*’‘ OR ''project*'' OR ‘‘program*’‘ OR ‘‘educat*’‘ OR ‘‘prevent*’‘ OR ‘‘promot*’‘ OR ‘‘counsel*’‘ OR ‘‘strateg*’‘ OR ‘‘communicat*’‘ OR ‘‘service*’‘ ) )

OR (KP= (‘‘pilot NEAR/1 study‘‘ OR ‘‘program NEAR/1 development ‘‘ OR ‘‘program NEAR/1 evaluation‘‘ OR ‘‘voluntary NEAR/1 program‘‘ OR ‘‘Internet-based NEAR/1 intervention‘‘ OR ‘‘sexual NEAR/1 education‘‘ OR ‘’health NEAR/1 promotion’’ OR ‘‘sex NEAR/1 counseling’‘))

OR (AK= (‘‘pilot NEAR/1 study‘‘ OR ‘‘program NEAR/1 development‘‘ OR ‘‘program NEAR/1 evaluation‘‘ OR ‘‘voluntary NEAR/1 program‘‘ OR ‘‘Internet-based NEAR/1 intervention‘‘ OR ‘‘sexual NEAR/1 education‘‘ OR ‘’health NEAR/1 promotion’’ OR ‘‘sex NEAR/1 counseling’‘))

(TI= (''abuse'' OR ''violence'' OR ''MSM'' OR ''YMSM'' OR ''men who have sex with men'' OR ''sex work*'' OR ''prostitut*'' or ''drug*'') ) OR (AB= (''abuse'' OR ''violence'') ) OR (KP= (''asia'' OR ''africa'' OR ''latin NEAR/1 america'' OR ''south NEAR/1 america'' OR ''central NEAR/1 america'' OR ''abuse'' OR ''violence'') ) OR (AK= (''asia'' OR ''africa'' OR ''latin NEAR/1 america'' OR ''south NEAR/1 america'' OR ''central NEAR/1 america'' OR ''abuse'' OR ''violence'') )

#3 AND #2 AND #1 AND PY=(2010-2021)

PsycInfo

(''condom*'' or ''safe* sex*'' or ''safe intercourse'' or ''unsafe sex*'' or ''unsafe intercourse'' or ''protected sex*'' or ''protected intercourse'' or ''unprotected sex*'' or ''unprotected intercourse'').ti. or (''condom*'' or ''safe* sex*'' or ''safe intercourse'' or ''unsafe sex*'' or ''unsafe intercourse'' or ''protected sex*'' or ''protected intercourse'' or ''unprotected sex*'' or ''unprotected intercourse'').ab. or (''condom*'' or ''safe sex'').mh. or (''condoms'' or ''safe sex'').sh.

(''young*'' or ''youth*'' or ''student*'' or ''teen*'' or ''school*'' or ''boy*'' or ''girl*'' or ''aya'' or ''adolesc*'').ti. or (''young*'' or ''youth*'' or ''student*'' or ''teen*'' or ''school*'' or ''boy*'' or ''girl*'' or ''aya'' or ''adolesc*'').ab. or (''young adult'' or ''adolescent'').mh.

(''intervention*'' or ''project*'' or ''program*'' or ''educat*'' or ''prevent*'' or ''promot*'' or ''counsel'' or ''strateg*'' or ''communicat*'' or ''service*'').ti. or (''intervention*'' or ''project*'' or ''program*'' or ''educat*'' or ''prevent*'' or ''promot*'' or ''counsel'' or ''strateg*'' or ''communicat*'' or ''service*'').ab. or (''pilot study'' or ''program development'' or ''program evaluation'' or ''voluntary program'' or ''Internet-based intervention'' or ''sexual education'' or ''health promotion'' or ''sex counseling'').mh. or (''pilot study'' or ''program development'' or ''program evaluation'' or ''voluntary program'' or ''Internet-based intervention'' or ''sexual education'' or ''health promotion'' or ''sex counseling'').sh.

(''abuse'' or ''violence'' or ''msm'' or ''ymsm'' or ''men who have sex with men'' or ''sex work*'' or ''prostitut*'' or ''drug*'').ti. or (''abuse'' or ''violence'').ab. or (''asia'' or ''africa'' or ''latin america'' or ''south america'' or ''central america'' or ''caribbean'' or ''abuse'' or ''violence'').mh. or (''asia'' or ''africa'' or ''latin america'' or ''south america'' or ''central america'' or ''abuse'' or ''violence'').sh.

(1 and 2 and 3) not 4

Limit 5 to yr=”2010-current”

de Vries, A., den Daas, C., Willemstein, I. J. M., de Wit, J. B. F., & Heijne, J. C. M. (2024). Interventions promoting condom use among youth: a systematic review. *Journal of Adolescent Health*, *74*(4), 644-656. <https://doi.org/https://doi.org/10.1016/j.jadohealth.2023.11.014>
